# Insanity Caused by Excessive Drinking, Followed by Anasarca

**Published:** 1844-11-16

**Authors:** 


INSANITY CAUSED BY EXCESSIVE DRINKING, FOLLOWED BY ANASARCA.

M. Brière de Boismont narrates, in the Gazette Medicale, the case of a foreigner, fifty years of age, of middle size, and lymphatic temperament, who had contracted a habit of drinking to excess, so that two or three bottles of brandy would be swallowed in a few minutes. These repeated excesses brought on an attack of delirium tremens eight years ago, which left a weakness of intellect. For some months past he required to be watched every moment; if he was lost sight of for a moment, he escaped, entered the first wine shop that presented itself, and left his watch, hat, or waistcoat, to pay his expenses. After one of these debauches, he was brought to M. Brière's establishment. His face was thin, his complexion yellow, his eyes dull, and without expression; tongue white, and no appetite. To all questions addressed to him, he replied "wine, brandy!" and put himself in a passion when he did not get what he wanted. At night he was quiet in bed; his step was firm, and there was no trembling in his limbs. The memory was weak, and he required to be told a thing twenty times before he could remember it. During the first three days he took little food, and coloured water was given him to drink. By degrees he got accustomed to the house, eat like the rest, and no longer asked for drink. Two months after his entrance, his appetite diminished daily. The face became lean, and assumed an earthy color: he remained motionless in a corner; and his legs became, soon afterwards, anasarcous. The horizontal position, nitrous drinks, and purgatives were prescribed, the infiltration began to diminish somewhat, and it then became stationary. The nitrate of potash in large doses and purgatives were given, and compression was also employed, thereby producing some amelioration. The loss of appetite, however, remained the same, and he continued to emaciate. His appearance gave indications of a serious organic attack. The mode of treatment hitherto pursued having produced little good, and the disease progressing, the dropsy was regarded as owing to deprivation of the habitual stimulants; consequently half-a-bottle of wine at each meal, with a glass of brandy, was prescribed. Scarcely had he been under this treatment eight days, when a most remarkable change was produced in his whole being. The effusion diminished, the physiognomy improved, the appetite returned, and, without having recourse to bandages, the swelling was radically cured in less than a fortnight. Isolation, and the moderate use of fermented liquors, had a favourable effect on his intellect. This patient, who had been the subject of complete dementia, a year after his admission could converse reasonably, although his ideas upon certain subjects were weakened. His memory, which seemed lost, eventually recovered almost all its strength. He related the events of his life, described the processes which he had observed in the arts, and desired to return to his family. One hallucination remained. Every day at three o'clock he saw his wife come into the room—she spoke to several persons in the house, but never addressed him. He has left the establishment quite cured; but it is pretty nearly certain that his *penchant* for spiritous liquors will occasion a relapse.—*London Medical Times.*

